# An R-CaMP1.07 reporter mouse for cell-type-specific expression of a sensitive red fluorescent calcium indicator

**DOI:** 10.1371/journal.pone.0179460

**Published:** 2017-06-22

**Authors:** Philipp Bethge, Stefano Carta, Dayra A. Lorenzo, Ladan Egolf, Despoina Goniotaki, Linda Madisen, Fabian F. Voigt, Jerry L. Chen, Bernard Schneider, Masamichi Ohkura, Junichi Nakai, Hongkui Zeng, Adriano Aguzzi, Fritjof Helmchen

**Affiliations:** 1 Brain Research Institute, University of Zurich, Zurich, Switzerland; 2 Neuroscience Center Zurich (ZNZ), University of Zurich and ETH Zurich, Zurich, Switzerland; 3 Institute of Neuropathology, University Hospital of Zurich, Zurich, Switzerland; 4 Allen Institute for Brain Science, Seattle, Washington, United States of America; 5 Brain Mind Institute, Ecole Polytechnique Fédérale de Lausanne (EPFL), Lausanne, Switzerland; 6 Saitama University, Brain Science Institute, Saitama, Japan; Universitatsklinikum Wurzburg, GERMANY

## Abstract

Genetically encoded calcium indicators (GECIs) enable imaging of in vivo brain cell activity with high sensitivity and specificity. In contrast to viral infection or in utero electroporation, indicator expression in transgenic reporter lines is induced noninvasively, reliably, and homogenously. Recently, Cre/tTA-dependent reporter mice were introduced, which provide high-level expression of green fluorescent GECIs in a cell-type-specific and inducible manner when crossed with Cre and tTA driver mice. Here, we generated and characterized the first red-shifted GECI reporter line of this type using R-CaMP1.07, a red fluorescent indicator that is efficiently two-photon excited above 1000 nm. By crossing the new R-CaMP1.07 reporter line to Cre lines driving layer-specific expression in neocortex we demonstrate its high fidelity for reporting action potential firing in vivo, long-term stability over months, and versatile use for functional imaging of excitatory neurons across all cortical layers, especially in the previously difficult to access layers 4 and 6.

## Introduction

Fluorescent activity sensors are widely used for measuring single-cell and neuronal population activity using various imaging techniques [1, 2]. Combined with two-photon microscopy such indicators allow monitoring of cellular activity relatively deep inside brain tissue of awake, behaving animals. Over the past 20 years, a multitude of genetically-encoded calcium indicators (GECIs) have been developed and continually improved, including single fluorescent protein sensors emitting in the green or red spectral range [3–11] as well as ratiometric sensors based on changes in fluorescence resonance transfer energy (FRET) between pairs of linked fluorescent proteins [12–17]. Classic labeling techniques such as bolus loading with synthetic calcium indicators [18] have been largely replaced by expression of one of the many available GECIs. Major advantages of GECIs are that they can be monitored chronically over weeks to months [4, 19–21] and that their expression can be targeted to specific cell types, allowing for imaging of defined neuronal subpopulations [3, 22–25]. For example, the Cre/lox recombination system, which relies on gene promoters or loci with specific expression patterns, is commonly used for genetic targeting of distinct cell types in mice [26–29].

Despite the progress in the design and application of GECIs, several challenges remain. First, in vivo calcium imaging requires relatively high GECI expression levels. Although good expression can be achieved by in utero electroporation or viral infection, these approaches have numerous drawbacks, including invasive surgical delivery, varying expression levels between neighboring cells, and incomplete coverage of targeted cell populations [3, 4]. Moreover, viral infection can cause cytotoxicity due to uncontrolled gene expression and/or long-term virus infection [3, 23]. To overcome these limitations, several transgenic mouse lines producing robust GECI-expression in selected tissues have been generated [30–35], including a Cre-dependent GCaMP3 and a tetracycline response element (TRE) dependent GCaMP7 mouse line for flexible genetic targeting [23, 36]. Although these transgenic line proved to be useful for some applications [37–40], limitations in sensitivity and functionality—presumably due to low expression levels—have been encountered in other situations [24]. Therefore, the Allen Institute of Brain Science, Seattle, established a new genomic locus, TIGRE, which allowed the generation of Cre- and tTA-dependent reporter lines with substantially increased expression levels [24]. Among a variety of suitable reporter lines for expression of functional sensors and effectors, mouse lines with strong and stable expression of GCaMP6s, GCaMP6f, and YCX2.60 were produced [24].

A second challenge is to achieve greater tissue depths for GECI imaging of neuronal activity (800 μm and beyond). Deep imaging, e.g., in infragranular neocortical layers or in subcortical structures, still remains a major goal of multi-photon microscopy [41, 42]. Because light scattering is reduced at longer wavelengths, calcium indicators with red-shifted two-photon excitation and fluorescence emission spectra can be advantageous for accessing deeper tissue regions. Consequently, many new red fluorescent calcium indicators have been developed, including synthetic dyes [43, 44] as well as GECIs [7, 8, 10, 17, 45, 46]. For example, R-CaMP1.07 was created by mutagenizing R-GECO1, introducing K47V and T49V mutations into the circular permutated domain of mApple as well as adding a C-terminal peptide F2A, which aids the nuclear export of the protein [7]. We recently showed that AAV-induced expression of R-CaMP1.07 enables calcium imaging in hippocampal dentate granule cells in awake mice [47], illustrating the possibility of deep imaging in the hippocampus. Additional exciting opportunities offered by red-shifted GECIs are dual-color functional imaging of distinct neuronal populations [6, 8] as well as the combination with optogenetics [6, 10].

Transgenic mouse lines expressing red-shifted GECIs have not been available so far. Here we report the generation and characterization of a Cre- and tTA-dependent TITL-R-CaMP1.07 reporter mouse (‘TIGRE-insulators-TRE promoter-LoxSTOPLox-R-CaMP1.07’) following the Allen Institute’s triple transgenic strategy. By combining this line with both tTA and layer-specific Cre driver lines we obtain R-CaMP1.07 expression specific to either neocortical layer 2/3 (L2/3), L4, L5 or L6, with stable indicator expression over months. We demonstrate efficient excitation of R-CaMP1.07 with fixed-wavelength lasers operating above 1000 nm and a high sensitivity of R-CaMP1.07 fluorescence signals for reporting action potential firing. In particular, neocortical layers previously difficult to study (L4 and L6) are amenable for calcium imaging with this new mouse line. Hence, the R-CaMP1.07 reporter mouse is a valuable addition to the collection of transgenic mouse lines designed for studying in vivo neuronal population activity.

## Results

### In vivo characterization of virally-expressed R-CaMP1.07

**Fig 1:**
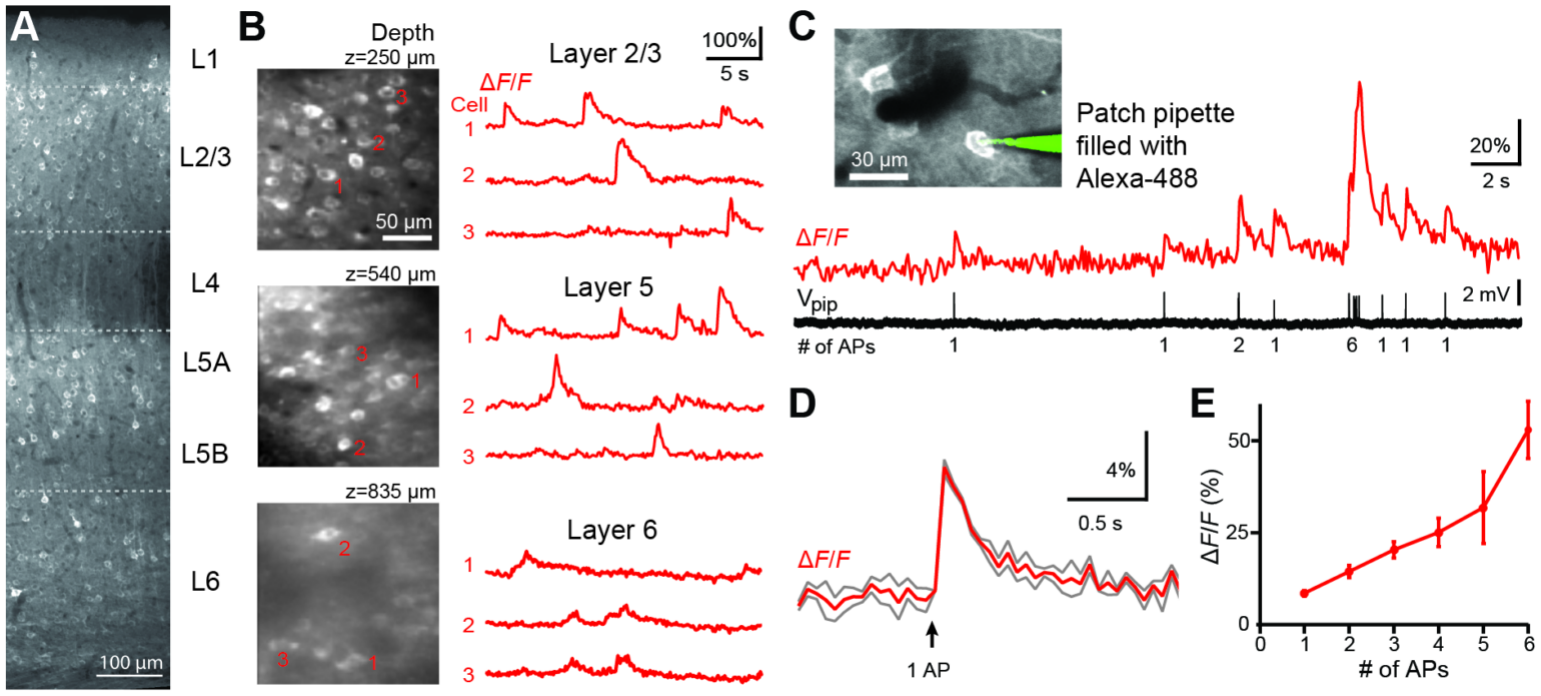
AAV-mediated viral expression of R-CaMP1.07 in vivo. **A,** Confocal image of a fixed brain slice illustrating R-CaMP1.07 expression across cortical layers of S1 barrel cortex after virus injections. **B,** Left: In vivo two-photon images of neurons expressing R-CaMP1.07 at different imaging depths down to L6. Right: Example spontaneous calcium transients in three example neurons (marked) per imaging field. **C,** Simultaneous fluorescence measurement and juxtacellular AP recording from an R-CaMP1.07-expressing L2/3 pyramidal neuron. Inset shows pipette attached to the cell. The Δ*F*/*F* trace shows calcium transients with variable amplitude, even resolving single-AP evoked transients. The number of spikes per burst is indicated below the voltage trace. **D,** Average Δ*F*/*F* calcium transient for R-CaMP1.07 in response to a single AP in red and (± S.E.M in grey; n = 49). **E**, Peak amplitudes of Δ*F*/*F* calcium transients as a function of the number of APs within short AP bursts (300-ms time window; mean ± S.E.M.).

To evaluate the suitability of R-CaMP1.07 for in vivo two-photon calcium imaging, we initially expressed R-CaMP1.07 in the neocortex of C57BL/6 mice using injections of AAV1-*EFα1-R-CaMP1*.*07* (Materials and methods). AAV injections resulted in neuronal expression of R-CaMP1.07 throughout all cortical layers except L4 (Fig 1A). Using two-photon microscopy, we measured spontaneous calcium transients of variable amplitude in L2/3, L5, and even L6 (Fig 1B). To assess the sensitivity of R-CaMP1.07 for reporting action potential (AP) firing, we performed juxtacellular recordings from R-CaMP1.07-expressing L2/3 neurons and quantitatively related calcium transients to simultaneously measured trains of APs (Fig 1C). In individual traces, single APs often, but not always, evoked clearly discernible calcium transients. On average, single-AP-evoked calcium transient displayed a Δ*F*/*F* peak amplitude of 7.4 ± 0.7% and a decay time constant of 0.28 ± 0.03 s (Fig 1D; n = 49 single-AP events in 3 cells; 2 mice). Calcium transient amplitude correlated with the number of spontaneous APs in bursts of APs (Fig 1E). We conclude that virally-expressed R-CaMP1.07 acts as a sensitive and fast red GECI suitable for deep in vivo calcium imaging.

### Generation of triple transgenic R-CaMP1.07 lines

**Fig 2:**
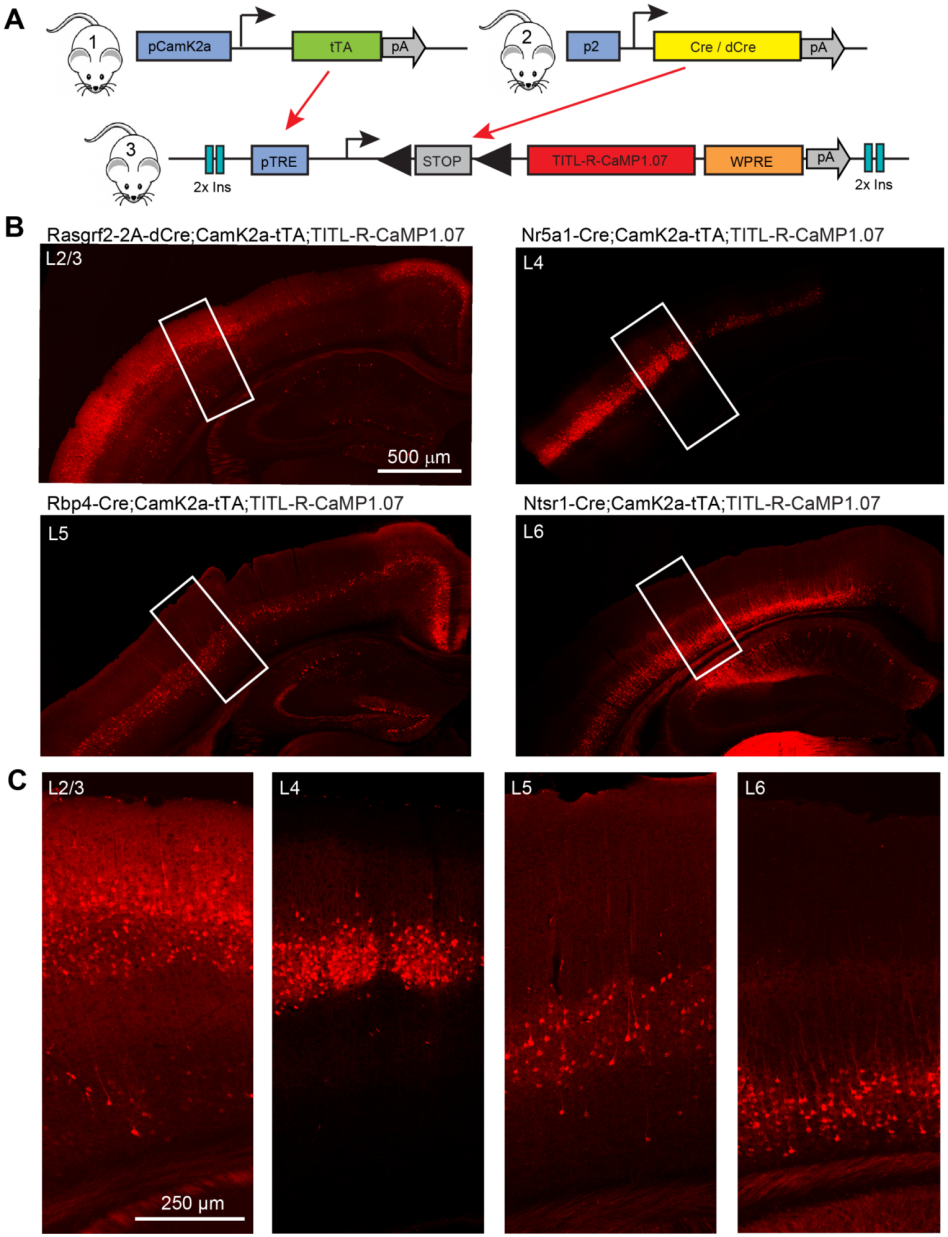
Characterization of layer-specific R-CaMP1.07 expression in triple transgenic mice. **A**, Schematic diagram of intersectional control by Cre and tTA, driven by different promoters, of the doubly regulated TITL-R-CaMP1.07 reporter line. tTA is driven by a CamK2a promoter, whereas layer-specificity is achieved by Cre expressed under the control of layer-specific promoters (p2: Rasgrf2-2A for L2/3, Nr5a1 for L4, Rbp4 for L5 and Ntsr1 for L6). **B**, Confocal images of coronal sections of fixed brains from the different mice, illustrating layer-specific labelling in neocortex of the respective mouse lines. **C**, High-resolution close-ups of the regions indicated in B, showing the R-CaMP1.07 expression patterns across cortical layers.

Encouraged by the good in vivo performance of R-CaMP1.07 we generated a Cre/tTA-dependent TITL-R-CaMP1.07 transgenic reporter mouse, which fits to the Allen Institute intersectional mouse lines [24]. To generate triple transgenic mice with layer-specific R-CaMP1.07 expression, we bred the TITL-R-CaMP1.07 mice with a CamK2a-tTA line [48] and one of four layer-specific Cre-driver lines (L2/3: Rasgrf2-2A-dCre; L4: Nr5a1-Cre; L5: Rbp4-Cre; L6: Ntsr1-Cre) (Fig 2A; Materials and methods). The resulting four triple transgenic R-CaMP1.07 mouse lines characterized in this study are: Rasgrf2-2A-dCre;CamK2a-tTA;TITL-R-CaMP1.07 (briefly ‘L2/3-R-CaMP1.07’), Nr5a1-Cre;CamK2a-tTA;TITL-R-CaMP1.07 (‘L4-R-CaMP1.07’), Rbp4-Cre;CamK2a-tTA;TITL-R-CaMP1.07 (‘L5-R-CaMP1.07’), and Ntsr1-Cre;CamK2a-tTA;TITL-R-CaMP1.07 (‘L6-R-CaMP1.07). Inspection of confocal image stacks of coronal histological sections of these triple transgenic mice confirmed R-CaMP1.07 expression specific to the respective cortical layer (Fig 2B). Several sub-cortical brain regions, e.g., hippocampal and thalamic regions, additionally expressed the R-CaMP1.07 indicator in some lines, in accordance with previous reports [24, 26, 49–51]. High-resolution confocal images illustrate widespread indicator expression in the targeted neuronal populations (Fig 2C). The L2/3-R-CaMP1.07 mouse showed prominent expression in neurons of L2/3, with a small number of additionally labeled cells scattered throughout the cortex. The L4-R-CaMP1.07 mouse showed exclusive expression in L4 whereas the L5-R-CaMP1.07 mouse exhibited prominent expression in L5A and L5B with additional neuronal labeling in the dentate gyrus and the CA1 region of the hippocampal formation. The L6-R-CaMP1.07 mouse expressed R-CaMP1.07 in pyramidal cells of neocortical L6 [52] as well as in some neurons of CA2 and CA1. In L5-R-CaMP1.07 and L6-R-CaMP1.07 mice, labeled apical dendrites were visible reaching L1 and L4, respectively. Overall, our histological analysis confirmed successful cross-sectional targeting of R-CaMP1.07 using the new reporter line.

### R-CaMP1.07 lines enable calcium imaging throughout neocortex

**Fig 3:**
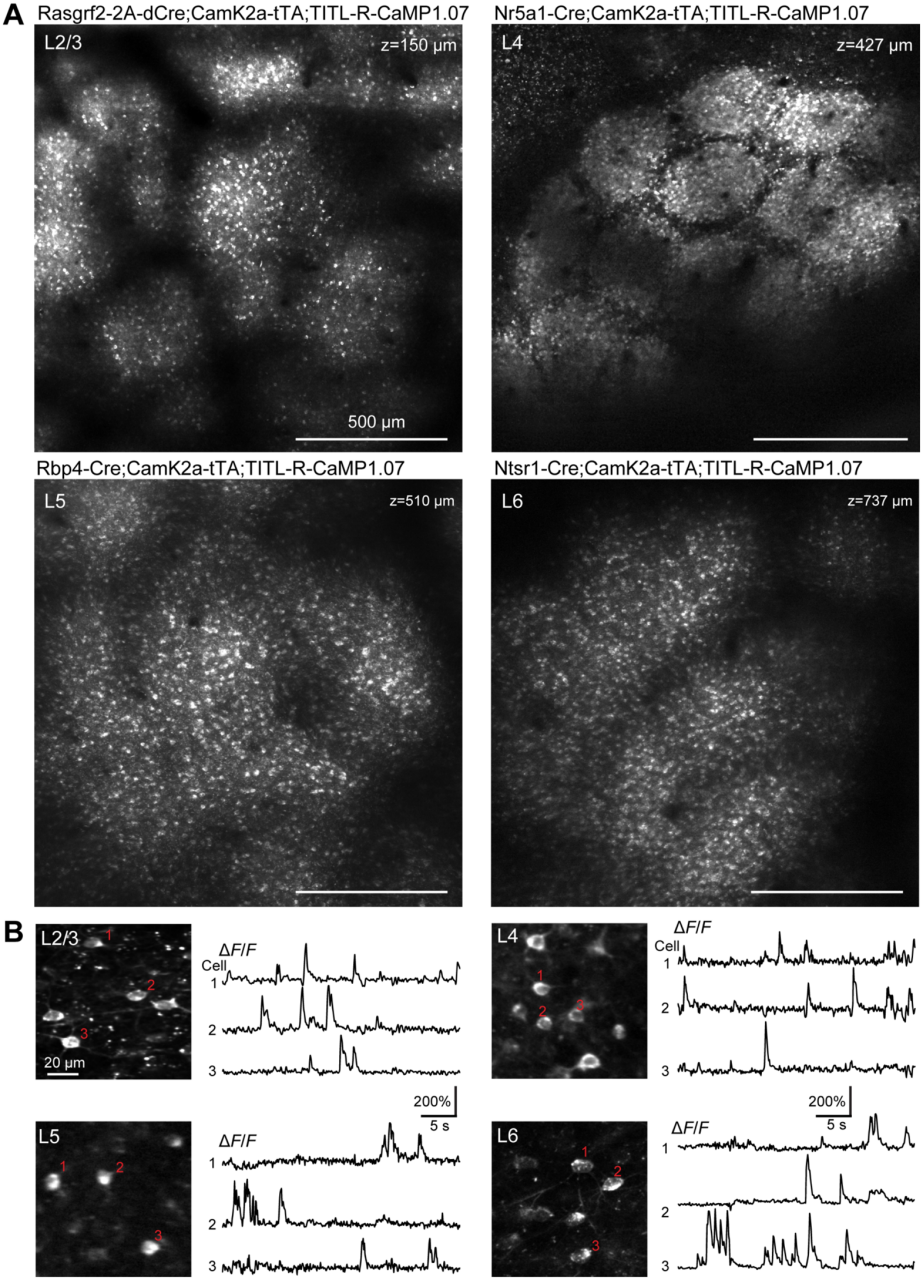
Layer-specific R-CaMP1.07 expression and calcium transients in vivo. **A,** Large field-of-view two-photon images in S1 cortex for the four different mouse lines expressing R-CaMP1.07. In L4-R-CaMP1.07 mice the barrels and septa in S1 cortex are clearly discernible. **B,** Example spontaneous R-CaMP1.07 calcium transients measured in awake mice of the corresponding mouse lines. Traces for three example cells (marked) are shown for each imaging field.

Next we aimed at characterizing R-CaMP1.07 expression pattern and its functionality in vivo. To assess neocortex-wide expression of R-CaMP1.07 in the respective layers, we implanted 5-mm diameter glass windows above the primary somatosensory cortex (S1) and measured R-CaMP1.07 signals using custom two-photon microscopes employing fixed-wavelength lasers operating at 1040 nm, 1044 nm and 1055 nm (Fig 3; Material and Methods). Aside of shadowing effects from surface blood vessels, indicator expression appeared uniform in the targeted cortical layer except for the L4-R-CaMP1.07 mouse, in which prominent barrels were clearly visible (Fig 3A). Images taken at higher zoom level revealed that the dense labeling of dendrites and axons of L4 stellate neurons, confined to the barrel column center and sparing the septa, caused this clear delineation of barrel cortex topography (S1 Fig). In L5-R-CaMP1.07 mice apical dendrites were clearly visible in the upper layers (S2 Fig). To a variable degree we observed bright fluorescent puncta in neuronal somata and in the neuropil. In the neuropil, puncta were not always simply cross-sectional views of dendrites and did not show time-variant signals. Linear unmixing using the relative spectral contribution of the puncta in the red and green emission channel upon 1044-nm excitation revealed that many of these structures were spectrally separable from R-CaMP1.07 labeling. In addition, we found similar puncta in L2/3 of a wild-type C57BL/6 mouse (<3 months old), indicating that at least partially these puncta represent unspecific autofluorescent structures rather than R-CaMP1.07 protein aggregates (S3 Fig). We also observed intrasomatic fluorescent aggregates, which possibly correspond to compartmentalized R-CaMP1.07 protein in lysosomes, similar to what has previously been reported for other R-GECO variants [11]. These intrasomatic puncta did not show measurable functional signals (S3 Fig).

**Fig 4:**
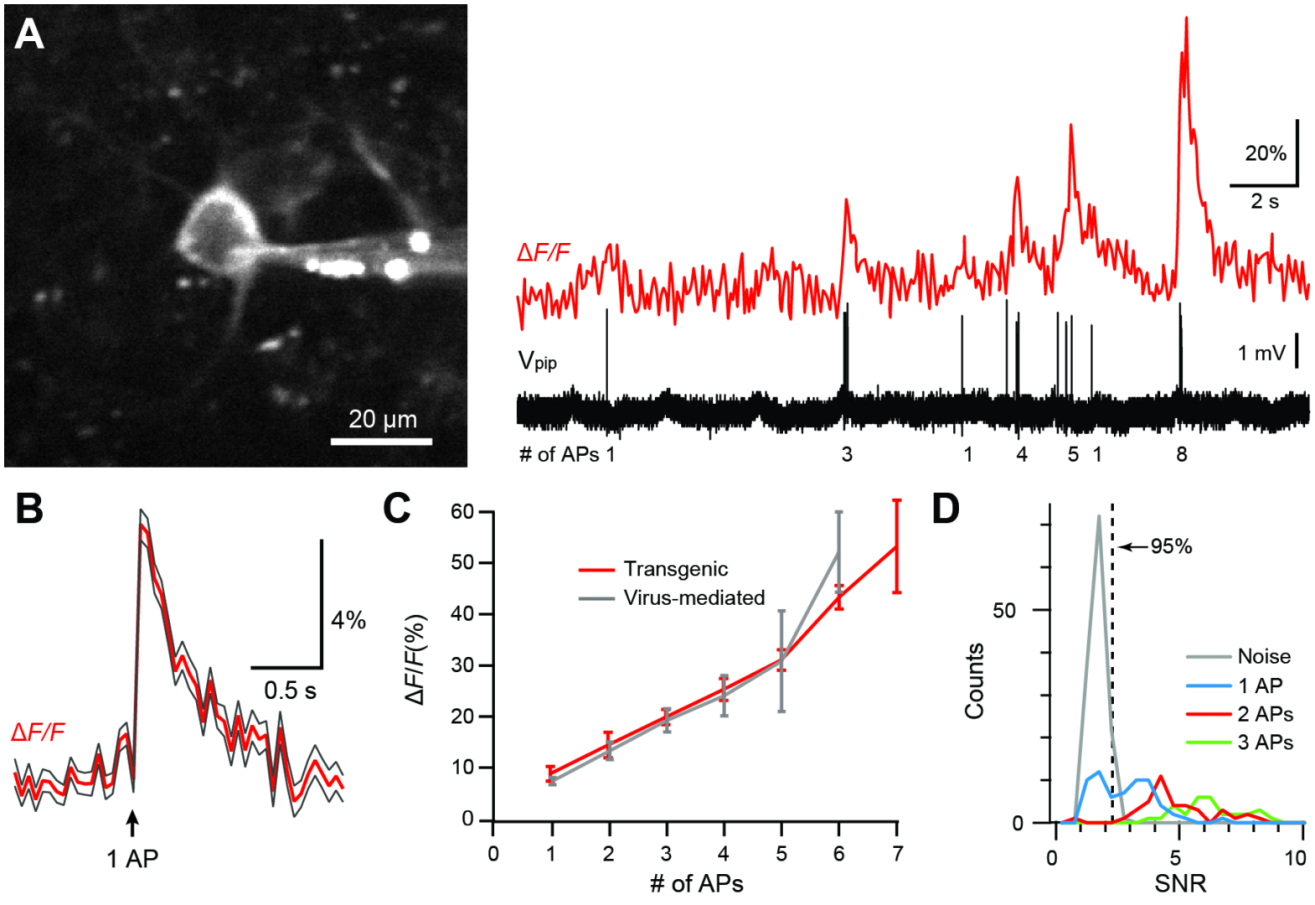
R-CaMP1.07 sensitivity for reporting APs in vivo. **A,** Left: Cell body of an R-CaMP1.07-expressing L2/3 neuron and recording pipette. Right: Simultaneous fluorescence measurement and juxtacellular AP recording from this neuron. The number of spikes per burst is indicated below the voltage trace. **B,** Average Δ*F*/*F* calcium transient for R-CaMP1.07 in response to a single AP in red (± S.E.M as grey traces; n = 63 transients from 8 cells, 3 mice). **C,** Peak amplitudes of Δ*F*/*F* calcium transients (red data points) as a function of the number of APs within short AP bursts (300-ms time window; mean ± S.E.M.). For comparison R-CaMP1.07 performance for AAV-mediated expression (same as Fig 1E) is overlaid (grey data points). **D,** Efficiency of AP detection in vivo was determined by estimating the distribution of the signal-to-noise ratio (SNR) under noise conditions and fitting with a Gaussian. From the fit, we determined the SNR cutoff at which less than 5% of baseline traces would be classified as false positives (SNR = 2.28). Using this threshold, 60% (38/63) of single APs, 97% of doublets (38/39) and 100% of triplets (30/30) were correctly detected (see ref. [53]).

In all R-CaMP1.07 mice we observed spontaneous somatic calcium transients under awake, head-restrained condition, reaching >200% Δ*F*/*F* amplitudes (Fig 3B). In the L5-R-CaMP1.07 line we were also able to observe rapid large calcium transients in the apical dendrites of labeled pyramidal neurons (S2 Fig). To compare R-CaMP1.07 performance in transgenic mice with AAV-induced expression, we performed in vivo juxtacellular recordings in anesthetized L2/3-R-CaMP1.07 mice. We found R-CaMP1.07 sensitivity for reporting AP firing in transgenic mice to be as high as for AAV-induced expression, sometimes clearly resolving single-AP-evoked fluorescence transients (Fig 4A and 4B). The average single-AP-evoked Δ*F*/*F* transient displayed an amplitude of 8.7 ± 1.4% and a decay time constant of 0.35 ± 0.07 s (Fig 4C; n = 63 events from 3 mice; both parameters are not significantly different from AAV-mediated R-CaMP1.07 expression; *P* = 0.19). Moreover, the relationship between Δ*F*/*F* transient amplitude and number of APs in brief bursts was nearly identical to the AAV-mediated expression condition (Fig 4C). In these experiments, the efficiency to detect single APs from the Δ*F/F* traces at 5% false positive rate was 60%, whereas doublet- and triplet-evoked calcium transients were detected with nearly 100% efficiency (Fig 4D). These results confirm a high fidelity of R-CaMP1.07 for reporting AP firing patterns with fast kinetics in transgenic mice, equivalent to in vivo conditions after virus injection (Fig 1) and in vitro conditions [7]. Furthermore, our demonstration of R-CaMP1.07 imaging of L6 neuronal activity highlights the benefit of red GECIs for deep cortical imaging.

### R-CaMP1.07 signals are stable over months

**Fig 5:**
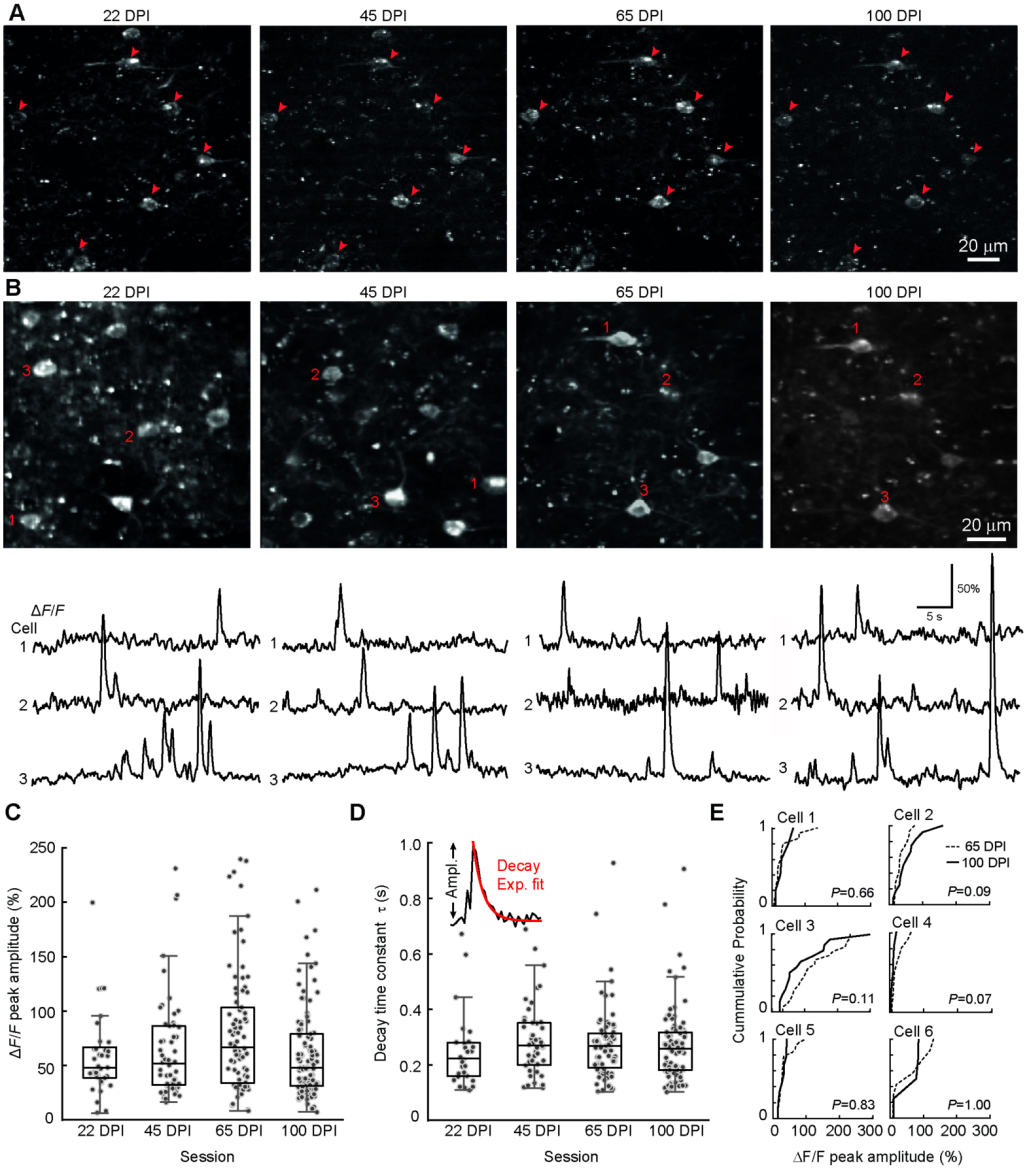
Long-term stability of L2/3 R-CaMP1.07 calcium signals. **A,** Longitudinal two-photon imaging of the same group of cells in L2/3 of S1 cortex in an example mouse, at 22, 45, 65 and 100 days post-induction (DPI). For a spectrally unmixed version of the image at 65 DPI see S3 Fig. **B,** Two-photon images of L2/3 neurons within the same tissue volume, for which R-CaMP1.07 calcium transients were measured across days. In the imaging sessions 65 DPI and 100 DPI the very same neurons were measured (same cells as in A). For each imaging area three example Δ*F*/*F* traces with spontaneous calcium transients are shown for the cells marked in the images above. **C**, Pooled analysis of stability of R-CaMP1.07 calcium transients. Recordings were made in 2 mice from a total of 18, 15, 15, and 38 active cells at 22, 45, 65, and 100 DPI, respectively. Data points and box plots of peak amplitudes of calcium transient events did not show significant variation across sessions. **D**, Data points and box plots of decay time constants (τ) of exponentially fitted calcium transients (see inset), which also did not show a significant change across imaging sessions. **E**, Cumulative distribution of calcium transient amplitudes for L2/3 neurons that were measured twice at 65 and 100 DPI, respectively. A non-parametric, Wilcoxon rank sum test was used to compute *P*-values.

Finally, we tested long-term stability of R-CaMP1.07 expression and functionality in L2/3 cells of S1 barrel cortex. Monitoring the same cells repeatedly for more than three months, we found that indicator expression was stable after reaching a plateau level during the initial 3 weeks following induction (Fig 5A and S4 Fig). Over the same time period, we observed in all sessions spontaneous calcium transient dynamics of L2/3 neurons (Fig 5B), with no significant differences of Δ*F/F* amplitudes and decay time constants, albeit amplitudes differed between the two mice analyzed (Fig 5C and 5D; amplitude: between mice *P* = 0.03, F_1,276_ = 4.81; across sessions *P* = 0.51, F_3,276_ = 0.77; decay time constant: between mice *P* = 0.68, F_1,262_ = 0.17; across sessions *P* = 0.37, F_3,262_ = 1.05; Nested ANOVA). In addition, the mean frequency of calcium transients in active neurons did not change significantly (22 ‘days post-induction’ [DPI]: 1.82 ± 1.94 events/min; 45 DPI: 2.12 ± 0.87 events/min; 65 DPI: 2.30 ± 1.17 events/min; 100 DPI: 2.22 ± 0.99 events/min; between mice *P* = 0.28, F_1,178_ = 1.3, across sessions *P* = 0.33, F_3,178_ = 0.33; n = 86 neurons; Nested ANOVA; the fraction of active cells was 23%, 25%, 62%, 42% at 22, 45, 65, and 100 DPI, respectively). Moreover, for a subset of cells we acquired functional data repeatedly across sessions, demonstrating that we could observe calcium transients over weeks in the very same neurons (Fig 5B and S5 Fig). For these cells the distribution of Δ*F/F* amplitudes of spontaneous events did not change over time (Fig 5E). We conclude that the R-CaMP1.07 reporter mouse provides stable expression and reliable functionality over at least 3 months.

## Discussion

We presented a novel mouse reporter line that specifically expresses the red calcium indicator R-CaMP1.07 in excitatory neurons of distinct cortical layers when crossed with the corresponding Cre line and tTA line and that reliably and sensitively reports AP-evoked calcium transients in the labeled neuronal populations. Key advantages of calcium imaging with red GECI mice are: (1) efficient two-photon excitation at wavelengths above 1000 nm, which allows imaging with cost-efficient fixed-wavelength lasers; and (2) reduced light scattering, which facilitates deep imaging as demonstrated here for cortical L6. By crossing the TITL-R-CaMP1.07 reporter line to various other Cre lines, we envision multifarious future applications for the study of neuronal activity patterns in awake, behaving mice.

In contrast to AAV-mediated viral expression of GECIs, expression of R-CaMP1.07 in the transgenic mouse is not restricted to the injection site and is uniform in the targeted neuronal subpopulation. For the RasGRF2-2A-dCre, Nr5a1-Cre, Rbp4-Cre and Ntsr1-Cre mouse lines tested here, we confirmed layer-specific expression throughout the entire cortical mantle. Interestingly, transgenic expression of R-CaMP1.07 using the Nr5a1-Cre line resulted in prominent labelling of L4 barrels in S1 cortex, which is a significant finding per se, because it has been difficult to infect L4 neurons using AAV-based viral approaches, hampering their study with in vivo calcium imaging so far. Moreover, the clear visibility of the L4 barrels in S1 cortex, presumably caused by the spatially confined high density of neural processes, opens great opportunities to relate functional properties of neurons and their processes to their precise anatomical location within the barrel cortex map.

The triple transgenic approach used here allows for long-term imaging (>10 weeks) of the same cells, which is of particular importance for studies requiring an extended observation period, such as for example studies on learning or training-intensive complex cognitive behaviors. Notably, calcium transient features remained stable for up to 3 months in the L2/3-R-CaMP1.07 mice, with no obvious degradation over time. However, we observed bright fluorescent puncta, especially with prolonged indicator expression in older mice. These kinds of structures have been previously described and were attributed to protein aggregation or accumulation in lysosomes [11, 30]. The exact origin of labeled puncta remains unclear, though. Given that we could spectrally separate many of them from R-CaMP1.07-expressing cell bodies and dendrites and also observed similar puncta in wildtype mice, they may, at least in part, represent autofluorescence of lipofuscin aggregates. These are known to accumulate with age in neurons and surrounding microglia [54–56]. Intrasomatic puncta with excitation/emission properties similar to R-CaMP1.07 did not show functional signals, hence slightly distorting Δ*F/F* values for somatic ROIs by adding largely calcium-insensitive fluorescence. However, their presence did not preclude the cells from showing cytosolic calcium transients of normal appearance.

The in vivo performance of R-CaMP1.07 reported here is comparable to R-CaMP2 [8] and jRGECO1a [11], which all are mApple-derived indicators as the original R-GECO [9]. In cortical neurons in vivo, these indicators exhibit calcium transients with about 10% Δ*F/F* changes for single APs and with 200–300 ms decay time constants. For R-CaMP1.07, we found similar AP sensitivity and Δ*F*/*F* transient kinetics for AAV-mediated as well as transgenic expression. Curiously, our data is not consistent with a previously reported inferior performance of R-CaMP1.07 compared to R-CaMP2 [8]. In general, mApple-derived red GECIs show the best performance in reporting AP firing so far, better compared to mPlum-derived indicators [11] and comparable with GCaMP6 indicators [4]. Moreover, they have lower Hill coefficients compared to GCaMPs, resulting in a reduction of non-linear responses to AP bursts [8]. A problematic feature of mApple-derived red GECIs is, however, that they consistently show photoswitching upon blue light stimulation, making them less suitable for combination with optogenetics [6, 10]. In the near future, further improved versions of red GECIs can be expected, which then might be considered for the generation of additional red GECI mouse lines.

The availability of red GECI mouse lines will open exciting further opportunities for the measurement of neuronal population activity. Combined with recent developments of novel cranial windows for extended optical access [57] and new large field-of-view two-photon microscopes with multi-regional imaging capabilities [58–61], red GECI mice could facilitate the visualization of distributed neuronal representations of various aspects of sensorimotor integration and task-related cortical processing across all cortical layers. Targeting specific cellular populations with a red GECI could also be combined with additional virus-induced expression of differently colored GECIs. Furthermore, the wide-spread, homogenous indicator expression in transgenic red GECI mice could be beneficial for wide-field camera imaging of dynamic activity maps, similar to experiments that have been conducted with transgenic mice expressing green GECIs as well as voltage and glutamate sensors [24, 62–64]. Potential inference of hemodynamic intrinsic signals will need to be carefully assessed, though [65]. In view of these prospects, we expect the TITL-R-CaMP1.07 reporter mouse line to be a valuable addition to the currently available set of GECI-expressing transgenic mice.

## Material and methods

### Ethics statement

All animal experimental procedures were carried out in strict accordance with the guidelines of the Veterinary Office of Switzerland and were approved by the Committee on the Ethics of Animal Experiments of the Cantonal Veterinary Office in Zurich (www.veta.zh.ch) under permit numbers 64/2012 and 285/2014. At the end of an experiment the animal was deeply anesthetized and transcardially perfused or euthanized with an overdose of pentobarbital (150 mg/kg body weight, i.p.). All efforts were made to minimize suffering.

### AAV vector production

The pAAV-EF1a-R-CaMP1.07 construct was obtained by subcloning the R-CaMP1.07 ORF from the original plasmid pN1-R-CaMP1.07 [7] into a AAV-EF1a-DIO-EYFP target vector (Addgene #27056) at BamHI and EcoRI sites. Recombinant serotype 1 AAV particles were produced in adherent AAV-293 cells by co-transfection with the pDF1 helper plasmid, using standard procedures. AAV1 particles were isolated from cell lysates using an iodixanol density gradient followed by anion exchange FPLC (GE Healthcare Bio-Sciences AB). Vector suspensions were concentrated in PBS using Centricon^®^ centrifugal filter devices with a molecular weight cut-off of 100 kDa (Millipore, Billerica, MA). For titration, the number of encapsidated genomes per ml was determined by real-time PCR.

### Transgenic mouse generation

The TITL-R-CaMP1.07 transgenic mouse line was generated following a flp-based recombinase-mediated cassette exchange (RMCE) strategy as described previously [24, 27]. The R-CaMP1.07 coding sequence, retrieved from the original plasmid pN1-G-CaMP1.07 generated in the Nakai lab [7], was cloned between the LSL cassette and the WPRE element in the targeting backbone to obtain a pTRE-LSL-R-CaMP1.07 replacement vector. This replacement vector contains (from 5' to 3') an FRT3 site, two copies of chicken beta-globin HS4 insulator element, a modified Tet response element (TRE), a LSL cassette, the R-CaMP1.07 coding sequence, a WPRE sequence, a bGH polyA, two copies of chicken beta-globin HS4 insulator element, an AttB site, a PGK-5'hygro cassette, an RNA splice donor and a FRT5 site. G4 embryonic stem cells previously targeted with FRT3::AttB::PGK-neoR polyA::FRT5::splice acceptor::3'hygro cassette::SV40 polyA:AttP to the TIGRE locus by homologous recombination [24], were re-transfected with a pTRE-LSL-R-CaMP1.07 replacement vector and a Flp recombinase vector for recombinase-mediated cassette exchange (RCME). Successful cassette exchange joined the 5'hygro cassette and 3'hygro cassette through mRNA splicing and reconstituted an active hygromycin-resistant gene, which could be used for selection of exchange events. Positive ES cell clones were injected into B6N-Tyrc blastocysts to obtain chimeric mice following standard procedures. Chimeric mice were bred with C57BL/6J mice. To identify transgenic animals Southern blot analysis was done on the F1 generation, using two external probes generated by PCR using the following primer pairs: 5’-tagggaagcactggccaaaggaa-3’, 5’-tcacggtaaccgcggcataaaac-3’ and 5’-cgaactgcccgctgttctgc-3’, 5’- gtagcgcgtctgctgctcca-3’. Transgenic mice were further crossed with C57BL/6J-congenic PhiC31 mice [66] to remove the AttB/AttP-flanked PGK-hygromycin-SV40polyA cassette. The resulting animals were crossed with C57BL/6J mice and their PhiC31 negative and R-CaMP1.07 positive progeny was used to establish the TITL-R-CaMP1.07 line. This line was bred heterozygously and the following primers were used for genotyping. R-CaMP1.07: 5’-caccatggtcgactcatcac-3’, 5’-acatgaactgaggggacagg-3’ (fragment length: 572 base pairs); wild type TIGRE locus: 5’-gtcaggcacaagaggtctacattc-3’, 5’-ggtggcacacacctttaatccc-3’ (fragment length: 380 base pairs). The TITL-R-CaMP1.07-D mouse line (Ai143D) is available from The Jackson Laboratory as JAX#030217.

### Mouse lines and animal handling

To generate triple transgenic animals, heterozygous double transgenic mice carrying tTA and R-CaMP1.07 were crossed with heterozygous Cre lines. The success rate in obtaining triple transgenic animals was 14%. Triple transgenic animals were healthy, fertile and did not show any gross phenotype. Breeding efficiency can be increased to 35% by crossing heterozygous triple transgenic animals from different breeding pairs with each other. However, this can lead to a slight decrease in litter size with an average of 5.5 animals per litter.

We used four Cre driver lines in this study, which were all crossed with CamK2a-tTA [48] and TITL-RCaMP1.07 mice to generate the animals used in this study: Rasgrf2-2A-dCre;CamK2a-tTA;TITL-R-CaMP1.07; Nr5a1-Cre;CamK2a-tTA;TITL-R-CaMP1.07; Rbp4-Cre;CamK2a-tTA;TITL-R-CaMP1.07; and Ntsr1-Cre;CamK2a-tTA;TITL-R-CaMP1.07. Each of these four lines contains a tet-off system, by which transgene expression can be suppressed upon doxycycline treatment [67, 68]. In this study we did not make use of this inducible system, which means that in the L4-, L5- and L6-R-CaMP1.07 lines, at the time of imaging, neurons were presumably expressing indicator already over several weeks, depending on the temporal profile of activity of the promoters driving tTA and Cre expression. As an alternative to the CamK2a-tTA mice, ROSA-ztTA mice could be used [48].

Note that the Rasgrf2-2A-dCre line holds an inducible system of its own, given that the destabilized Cre (dCre) expressed under the control of the Rasgrf2-2A promoter needs to be stabilized by trimethoprim (TMP) to be fully functional. In this study we used this induction system. TMP (Sigma T7883) was reconstituted in Dimethyl sulfoxide (DMSO, Sigma 34869) at a saturation level of 100 mg/ml, freshly prepared for each experiment. For TMP induction, mice were given a single intraperitoneal injection (150 μg TMP/g body weight; 29g needle), diluted in 0.9% saline solution.

For long-term two-photon calcium imaging in L2/3, 2-month old L2/3-R-CaMP1.07 mice (n = 4) were used, starting imaging 10 days post-induction (DPI). Two of these mice were followed up with repeated imaging up to 110 DPI. For R-CaMP1.07 virus injections 4–6 weeks old C57BL/6J mice were used (n = 3). Mice were housed 2–3 per cage under reverse light/dark cycle (12h/12h) conditions. All handling and behaviour experiment occurred under simulated night time conditions. For awake experiments, mice were accustomed to head-fixation during one week of daily handling and increasing duration of head fixation.

### Histology and confocal imaging

Mice were deeply anesthetized (ketamine/xylazine; 100/20 mg/kg body weight) and perfused transcardially with 4% paraformaldehyde in phosphate buffer, pH 7.4. Coronal sections (300 μm) were cut from the fixed brains using a vibratome (VT100; Leica) and mounted (Vectashield). Images of R-CaMP1.07 expression patterns were acquired with a confocal microscope (Fluoview 1000; Olympus, tdTomato excitation/emission filters) using a motorised stage for mosaic acquisition (UPLSAPO20X objective) of large field of views.

### Surgical procedures and viral injections

For virus-induced expression of R-CaMP1.07 in neurons of barrel cortex, AAV1-*EFα1-R-CaMP1*.*07* was stereotactically injected through glass micropipettes in 4–6 weeks old C57BL/6J mice under isoflurane anesthesia as described previously [69]. Multiple injections of a total volume of 200–300 nl of virus-containing solution were made throughout cortical layers (100–800 μm depth). To allow long-term in vivo calcium imaging, a 3-mm diameter cranial window was implanted over S1 cortex in transgenic mice as described previously [69]. A metal post for head fixation was implanted on the skull contralateral to the cranial window using dental acrylic cement.

### Two-photon calcium imaging

Custom-built two-photon microscopes were used for imaging of R-CaMP1.07 employing various ultrafast laser (Figs 1, 4, and 5, and S4 Fig: 1040-nm excitation, 229-fs pulse width, 80-MHz repetition rate, YBIX, Lumentum, USA [formerly Time-Bandwidth Products, Zurich, Switzerland]; Fig 3A and S1 and S3 Figs: 1044-nm excitation, 270-fs pulse width, 21-MHz repetition rate, Femtotrain 1040–2000, SpectraPhysics, USA [formerly High-Q Laser GmbH, Rankweil, Austria]; Fig 3B, S2 Fig: 1055-nm, 80-fs pulse width, 80-MHz repetition rate, Origami-10HP, OneFive, Zurich, Switzerland). A 16x/0.80NA water objective was employed (Nikon N16XLWD-PF) except for Fig 3A, for which a 10x-objective was used (Olympus XLPlan N 10x/0.6NA) and S1 Fig (panel B), for which a 20x-objective was used (XLUMPlanFl 20X/0.95NA). Standard galvanometric scan mirrors were employed except for the measurements of the calcium transients in L2/3, L4 and L5 in Fig 3B and S2 Fig, for which a 4-kHz resonant scanner was used. For Fig 3 and for S1 and S3 Figs the fluorescence emission signal was directed towards a hybrid-photo-detector (R11322U-40 MOD, Hamamatsu; Preamplifier C1077B, Hamamatsu) via a dichroic mirror (KS93, Qioptic), before passing a blocking filter (FF01-720/SP-25, Semrock) and, an emission filter (Chroma ET 605/70 M). The signal was digitized by an analog-to-digital converter (ADC, NI-5771, National Instruments) connected to a field-programmable array (FPGA, NI-7962R, National Instruments). For microscope control, the custom-written C++ software ‘SCOPE’ was used (Visual Studio C++; see http://rkscope.sourceforge.net/). For Figs 1, 4, 5 and S4 Fig, a Sutter Instrument Movable Objective Microscope was used, which was controlled by HelioScan (Langer et al., 2013).

For longitudinal assessment of stability, L2/3 neurons in S1 cortex were repeatedly imaged in head-restrained, awake mice starting at 1 DPI, and up to 100 DPI (in some animals up to 110 DPI). In each imaging session, we acquired stacks of 256x256 pixel images starting at the brain surface down to 330-μm depth (frame rate 12 Hz; 5-frames averaged per image; Δz = 3 μm). To ensure that we imaged the same tissue volume across sessions, we used surface blood vessel pattern and salient L2/3 cell groups as landmarks. Once full expression of the indicator was reached (around 22 DPI) we also imaged spontaneous activity in subsets of neurons within the same volume across sessions (22 DPI, 45 DPI, 65 DPI, and 100 DPI; 12-Hz frame rate).

### Combined juxtacellular recordings and two-photon imaging

Combined electrophysiology and in vivo calcium imaging was performed in acute experiments in virus-infected mice (n = 3; at least two weeks after injection) and in L2/3-R-CaMP1.07 mice (n = 3). Mice were anesthetized with isoflurane (5% for induction) and temperature was maintained at 35°C during the surgery with a heating pad (Watlow). During imaging, body temperature was maintained at 37°C and the anesthesia level was lowered (2%) for increasing cortical activity. A stainless steel plate was fixed to the exposed skull by using dental acrylic cement. A 1x1 mm^2^ craniotomy was performed over barrel cortex and the dura was cleaned with Ringer’s solution (containing in mM: 135 NaCl, 5.4 KCl, 1.8 CaCl_2_, 5 HEPES, pH 7.2 with NaOH) and carefully removed. To reduce tissue motion caused by heart beat and breathing, the craniotomy was filled with low concentration agarose gel and gently pressed with a glass coverslip.

Juxtacellular recordings from R-CaMP1.07-expressing L2/3 neurons were obtained with glass pipettes (4–6 MΩ tip resistance) containing Ringer’s solution. For pipette visualization Alexa-488 (Invitrogen) was added to the solution or pipettes were coated with BSA Alexa-594 (Invitrogen). Action potentials were recorded in current clamp at 10-kHz sampling rate using an Axoclamp 2-B amplifier (Axon Instruments, Molecular Devices) and digitized using Clampex 10.2 software.

### Data analysis

Analysis was performed using ImageJ for image analysis and custom-written MATLAB scripts. Regions of interests (ROIs) corresponding to individual neurons were manually selected. Calcium signals were expressed as relative percentage change Δ*F*/*F* = (*F*-*F*_0_)/*F*_0_, with *F*_0_ was calculated for each trace as the bottom 8th percentile of the background-subtracted fluorescence distribution. Neurons were considered active if they showed events in the Δ*F*/*F* traces reaching levels higher than 3 standard deviations above the median; each of these events was considered a calcium transient, most of them presumably reflecting bursts of APs. Peak amplitude of each calcium transient was calculated as the 3-point average around the maximum value and an exponential function was fitted to the calcium transient decay time course from the maximum point over a 1.1-s window, yielding the decay time constant τ. To test stability of these functional properties (amplitude and decay time constant) over time, we performed a one-way ANOVA on the data pooled for all active neurons across the four measured time points (22, 45, 65, and 100 DPI, respectively).

Peak amplitudes of AP-evoked Δ*F*/*F* transients were calculated as the average of three sample points around the peak location. The time window of including APs to a calcium transient was defined to be 300 ms. Decay time constant was determined by fitting am exponentially decaying function to the average calcium transient evoked by individual spontaneously occurring APs. We estimated the saturating maximal Δ*F/F* change for R-CaMP1.07 (Δ*F*/*F*_max_) at the end of several juxtacellular recordings by injecting a large current through the recording pipette, yielding 245 ± 12% (mean ± S.E.M.; n = 7 cells) and 373 ± 33% (n = 11), for virus-induced and transgenic expression, respectively. These values are rough and likely to still underestimate the true value, because Δ*F*/*F* values are sensitive to resting calcium concentration, background estimation and *F*_0_ calculation.

To quantify AP detection efficiency, the signal-to-noise (SNR) of calcium transients was calculated as the ratio of peak amplitude to the standard deviation of the unfiltered Δ*F/F* trace in a 1-s baseline time window prior to the first AP. To determine the percentage of detectable transients with less than 5% false positives, a threshold was set at the 95^th^ percentile of the distribution of baseline SNRs, which was calculated as the ratio of the peak to the SD of the respective baseline trace. The fraction of detectable calcium transients evoked by individual APs and AP bursts within 300-ms windows, respectively, were determined as the fraction of traces with SNR above this threshold [53].

Linear unmixing of the emission signals collected via a green (Brightline Basic 535/22) and a red (Chroma ET 605/70 M) emission filter was performed using the Spectral Unmixing plugin for ImageJ (Walter, J.; see https://imagej.nih.gov/ij/plugins/spectral-unmixing.html).

## Supporting information

S1 Fig High-resolution imaging of whisker barrels in S1 cortex. **A,** High-resolution two-photon image of R-CaMP1.07-expressing neurons in L4 of S1 barrel cortex in a L4-R-CaMP1.07 mouse. An individual barrel is clearly visible (outline highlighted by dashed line) using a Nikon 16x NA0.8 objective. **B**, The diffuse labelling within the barrel is further resolved as labeled neuropil structures using a Olympus 20x NA1.0 objective. Different barrel as in A.
(PDF)

S2 Fig Dendritic calcium imaging of L5 neurons. **A,** Two-photon image of L5 pyramidal dendrite cross-sections at the level of L4 in a L5-R-CaMP1.07 mouse (average image from a time series acquired at 12.3 Hz frame rate). **B**, Example spontaneous R-CaMP1.07 calcium traces in individual dendritic cross-sections marked in A. **C**, X-Z view created from an image stack acquired in a lightly anesthetized L5-R-CaMP1.07 mouse. **D**, Top: Single-plane images at different cortical depths (indicated by arrows in C); Bottom: Example R-CaMP1.07 fluorescence transients measured in cell somata (ROIs 1–4) and dendrites (ROIs 5–12) at the different imaging depths.
(PDF)

S3 Fig Lipofuscin-like puncta in transgenic and wildtype mice. **A,** In both C57BL/6 wild-type mice (top) and L2/3-R-CaMP1.07 mice (bottom), small puncta can be found throughout the cortex. Note that no additional labelling was introduced. **B**, Puncta emit differentially in the red and the green channel when excited at 1044 nm and can therefore be unmixed using a spectral unmixing plugin for ImageJ. The color-merged overlay of the unmixed images thus discriminates autofluorescent puncta from dendritic cross-sections. **C**, Another example of unmixing for an image acquired in a L2/3-R-CaMP1.07 mouse. The image corresponds to the field-of-view shown in the third session of the longitudinal experiments shown in Fig 5A. **D**, Analysis of fluorescence changes of intrasomatic puncta. Left: Two-photon image of neurons in a L2/3-R-CaMP1.07 mouse. For three neurons small ROIs surrounding intrasomatic puncta were selected as well as a cytosolic ROI sparing these puncta. Two additional extrasomatic puncta (13 and 14) in the surrounding tissue were selected, too. Right: Time course of Δ*F/F* traces for the selected ROIs. Note the large spontaneous calcium transients in the cytosol of two neurons. Except for the dim punctum 7, which may be blurred by cytosolic signal, puncta did not show detectable calcium signals.
(PDF)

S4 Fig Post-induction time course of R-CaMP1.07 expression. Repeated imaging of R-CaMP1.07 expression in an example L2/3-R-CaMP1.07 mouse following TMP induction (different mouse from Fig 5A). On the top left overview images of the chronic cranial window of 10 and 50 DPI are shown. R-CaMP1.07 expressing cells were imaged at 10, 15, 22, 30, 40 and 50 DPI. First R-CaMP1.07 expression could be observed at 10 DPI. Longitudinal imaging of the same group of 11 cells in L2/3 of S1 cortex (indicated by arrows) could be performed from 22 DPI onwards.
(PDF)

S5 Fig Repeated functional imaging of R-CaMP1.07 signals from L5 neurons. Top: Two-photon images of example L5 neurons in a L5-R-CaMP1.07 mouse taken two weeks apart. Bottom: Repeatedly measured spontaneous somatic Δ*F/F* calcium signals for the 4 example neurons marked in the images.
(PDF)
