# Supplementary material for: An R-CaMP1.07 reporter mouse for cell-type-specific expression of a sensitive red fluorescent calcium indicator
Source: PLoS One. 2017 Jun 22;12(6):e0179460. doi: 10.1371/journal.pone.0179460 (PMC5480891; doi:10.1371/journal.pone.0179460)
Supplement: S1 Fig — A, High-resolution two-photon image of R-CaMP1.07-expressing neurons in L4 of S1 barrel cortex in a L4-R-CaMP1.07 mouse. An individual barrel is clearly visible (outline highlighted by dashed line) using a Nikon 16x NA0.8 objective. B, The diffuse labelling within the barrel is further resolved as labeled neuropil structures using a Olympus 20x NA1.0 objective. Different barrel as in A. (PDF) [file pone.0179460.s001.pdf]

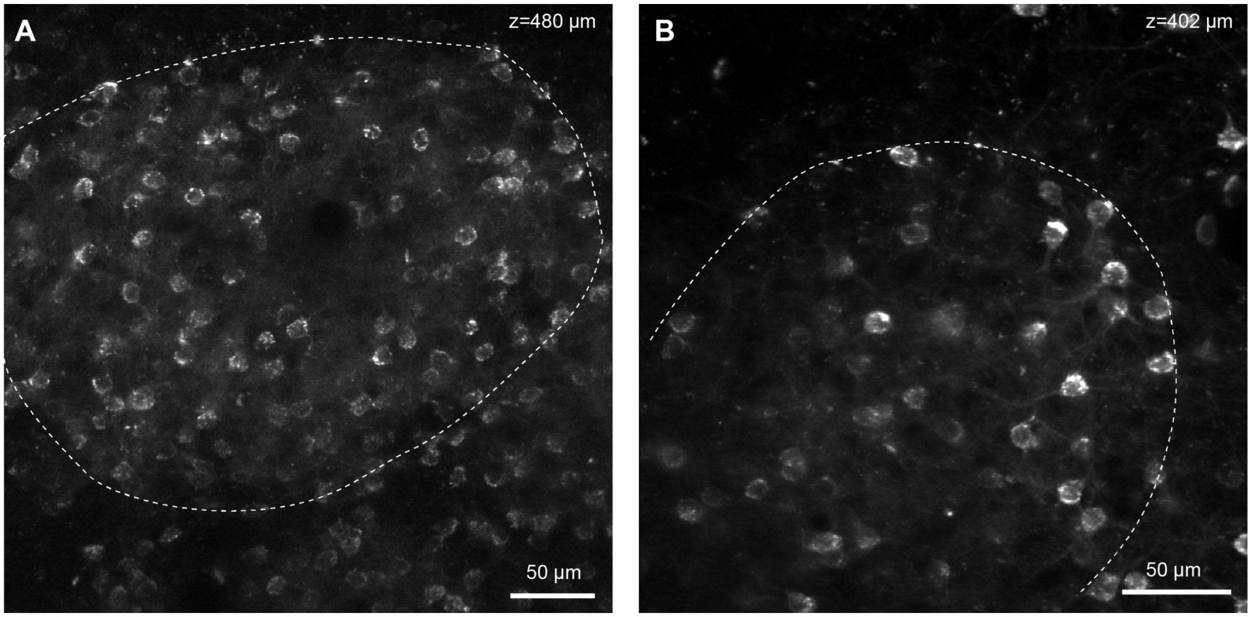

S1 Fig. High-resolution imaging of whisker barrels in S1 cortex. A, High-resolution two-photon image of R-CaMP1.07-expressing neurons in L4 of S1 barrel cortex in a L4-R-CaMP1.07 mouse. An individual barrel is clearly visible (outline highlighted by dashed line) using a Nikon 16x NA0.8 objective. **B**, The diffuse labelling within the barrel is further resolved as labeled neuropil structures using a Olympus 20x NA1.0 objective. Different barrel as in A.
