# Supplementary material for: An R-CaMP1.07 reporter mouse for cell-type-specific expression of a sensitive red fluorescent calcium indicator
Source: PLoS One. 2017 Jun 22;12(6):e0179460. doi: 10.1371/journal.pone.0179460 (PMC5480891; doi:10.1371/journal.pone.0179460)
Supplement: S2 Fig — A, Two-photon image of L5 pyramidal dendrite cross-sections at the level of L4 in a L5-R-CaMP1.07 mouse (average image from a time series acquired at 12.3 Hz frame rate). B, Example spontaneous R-CaMP1.07 calcium traces in individual dendritic cross-sections marked in A. C, X-Z view created from an image stack acquired in a lightly anesthetized L5-R-CaMP1.07 mouse. D, Top: Single-plane images at different cortical depths (indicated by arrows in C); Bottom: Example R-CaMP1.07 fluorescence transients measured in cell somata (ROIs 1–4) and dendrites (ROIs 5–12) at the different imaging depths. (PDF) [file pone.0179460.s002.pdf]

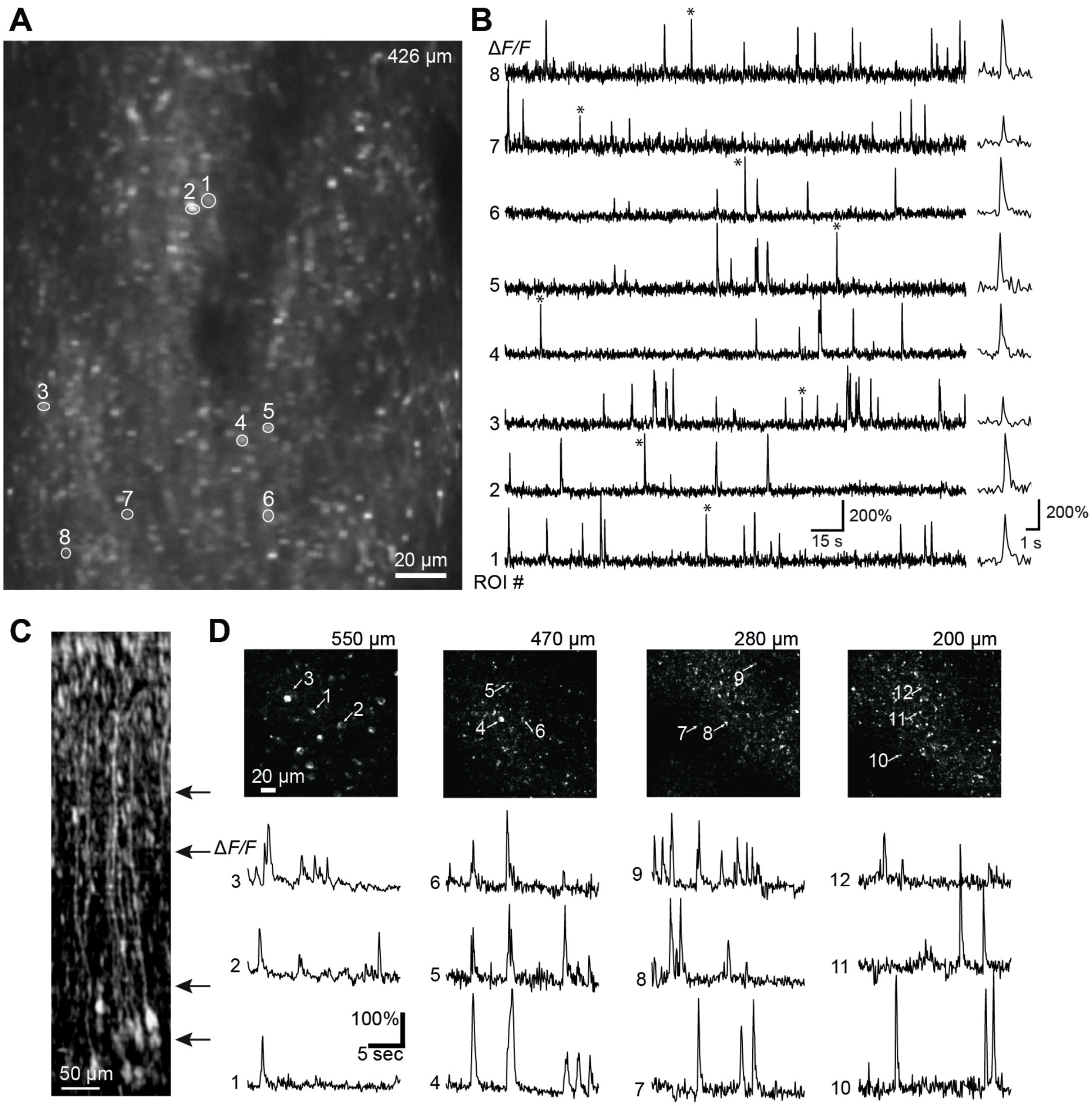

S2 Fig. Dendritic calcium imaging of L5 neurons. A, Two-photon image of L5 pyramidal dendrite cross-sections at the level of L4 in a L5-R-CaMP1.07 mouse (average image from a time series acquired at 12.3 Hz frame rate). **B**, Example spontaneous R-CaMP1.07 calcium traces in individual dendritic cross-sections marked in A. **C**, X-Z view created from an image stack acquired in a lightly anesthetized L5-R-CaMP1.07 mouse. **D**, Top: Single-plane images at different cortical depths (indicated by arrows in C); Bottom: Example R-CaMP1.07 fluorescence transients measured in cell somata (ROIs 1-4) and dendrites (ROIs 5-12) at the different imaging depths.
