# Supplementary material for: An R-CaMP1.07 reporter mouse for cell-type-specific expression of a sensitive red fluorescent calcium indicator
Source: PLoS One. 2017 Jun 22;12(6):e0179460. doi: 10.1371/journal.pone.0179460 (PMC5480891; doi:10.1371/journal.pone.0179460)
Supplement: S3 Fig — A, In both C57BL/6 wild-type mice (top) and L2/3-R-CaMP1.07 mice (bottom), small puncta can be found throughout the cortex. Note that no additional labelling was introduced. B, Puncta emit differentially in the red and the green channel when excited at 1044 nm and can therefore be unmixed using a spectral unmixing plugin for ImageJ. The color-merged overlay of the unmixed images thus discriminates autofluorescent puncta from dendritic cross-sections. C, Another example of unmixing for an image acquired in a L2/3-R-CaMP1.07 mouse. The image corresponds to the field-of-view shown in the third session of the longitudinal experiments shown in Fig 5A. D, Analysis of fluorescence changes of intrasomatic puncta. Left: Two-photon image of neurons in a L2/3-R-CaMP1.07 mouse. For three neurons small ROIs surrounding intrasomatic puncta were selected as well as a cytosolic ROI sparing these puncta. Two additional extrasomatic puncta (13 and 14) in the surrounding tissue were selected, too. Right: Time course of ΔF/F traces for the selected ROIs. Note the large spontaneous calcium transients in the cytosol of two neurons. Except for the dim punctum 7, which may be blurred by cytosolic signal, puncta did not show detectable calcium signals. (PDF) [file pone.0179460.s003.pdf]

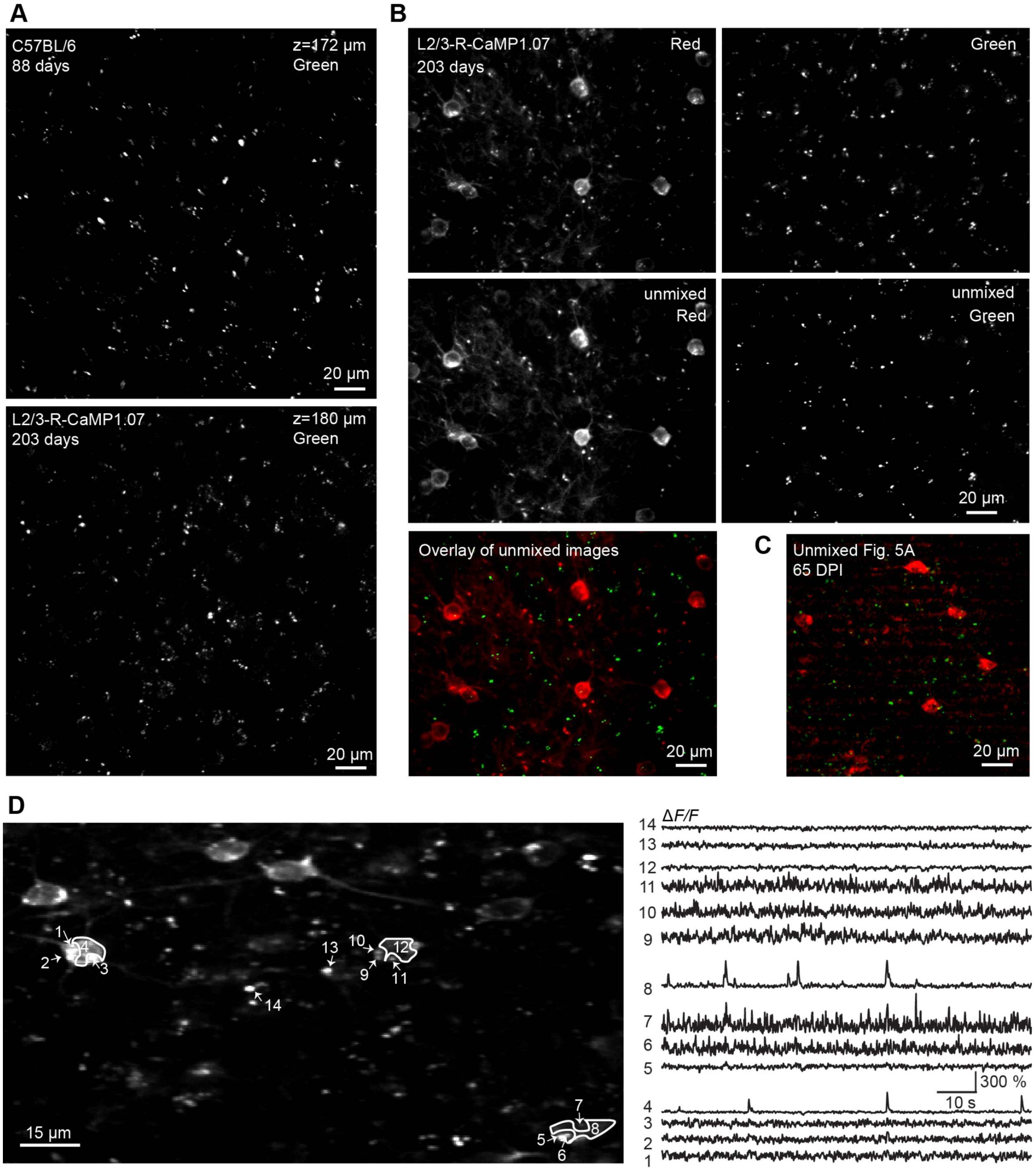

S3 Fig. Lipofuscin-like puncta in transgenic and wildtype mice.

A, In both C57BL/6 wild-type mice (top) and L2/3-R-CaMP1.07 mice (bottom), small puncta can be found throughout the cortex. Note that no additional labelling was introduced. **B**, Puncta emit differentially in the red and the green channel when excited at 1044 nm and can therefore be unmixed using a spectral unmixing plugin for ImageJ. The color-merged overlay of the unmixed images thus discriminates autofluorescent puncta from dendritic cross-sections. **C**, Another example of unmixing for an image acquired in a L2/3-R-CaMP1.07 mouse. The image corresponds to the field-of-view shown in the third session of the longitudinal experiments shown in Fig. 5A. **D**, Analysis of fluorescence changes of intrasomatic puncta. Left: Two-photon image of neurons in a L2/3-R-CaMP1.07 mouse. For three neurons small ROIs surrounding intrasomatic puncta were selected as well as a cytosolic ROI sparing these puncta. Two additional extrasomatic puncta (13 and 14) in the surrounding tissue were selected, too. Right: Time course of Δ*F/F* traces for the selected ROIs. Note the large spontaneous calcium transients in the cytosol of two neurons. Except for the dim punctum 7, which may be blurred by cytosolic signal, puncta did not show detectable calcium signals.
