# Supplementary material for: An R-CaMP1.07 reporter mouse for cell-type-specific expression of a sensitive red fluorescent calcium indicator
Source: PLoS One. 2017 Jun 22;12(6):e0179460. doi: 10.1371/journal.pone.0179460 (PMC5480891; doi:10.1371/journal.pone.0179460)
Supplement: S4 Fig — Repeated imaging of R-CaMP1.07 expression in an example L2/3-R-CaMP1.07 mouse following TMP induction (different mouse from Fig 5A). On the top left overview images of the chronic cranial window of 10 and 50 DPI are shown. R-CaMP1.07 expressing cells were imaged at 10, 15, 22, 30, 40 and 50 DPI. First R-CaMP1.07 expression could be observed at 10 DPI. Longitudinal imaging of the same group of 11 cells in L2/3 of S1 cortex (indicated by arrows) could be performed from 22 DPI onwards. (PDF) [file pone.0179460.s004.pdf]

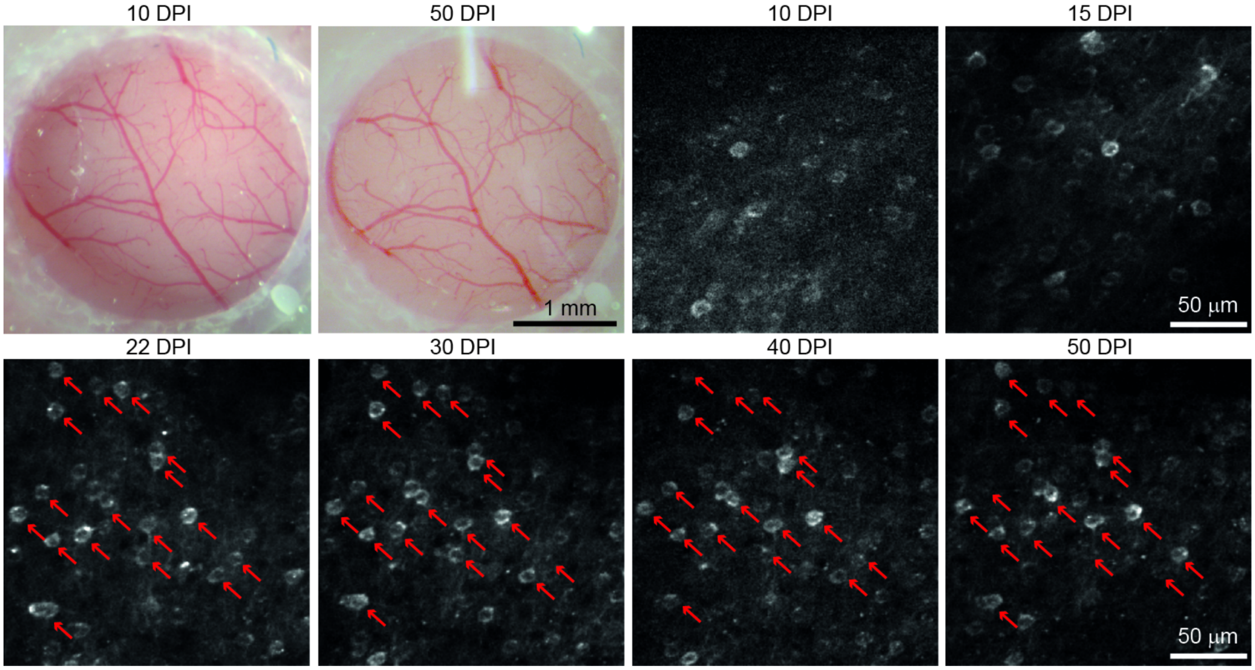

S4 Fig. Post-induction time course of R-CaMP1.07 expression. Repeated imaging of R-CaMP1.07 expression in an example L2/3-R-CaMP1.07 mouse following TMP induction (different mouse from Fig 5A). On the top left overview images of the chronic cranial window of 10 and 50 DPI are shown. R-CaMP1.07 expressing cells were imaged at 10, 15, 22, 30, 40 and 50 DPI. First R-CaMP1.07 expression could be observed at 10 DPI. Longitudinal imaging of the same group of 11 cells in L2/3 of S1 cortex (indicated by arrows) could be performed from 22 DPI onwards.
