# Supplementary material for: An R-CaMP1.07 reporter mouse for cell-type-specific expression of a sensitive red fluorescent calcium indicator
Source: PLoS One. 2017 Jun 22;12(6):e0179460. doi: 10.1371/journal.pone.0179460 (PMC5480891; doi:10.1371/journal.pone.0179460)
Supplement: S5 Fig — Top: Two-photon images of example L5 neurons in a L5-R-CaMP1.07 mouse taken two weeks apart. Bottom: Repeatedly measured spontaneous somatic ΔF/F calcium signals for the 4 example neurons marked in the images. (PDF) [file pone.0179460.s005.pdf]

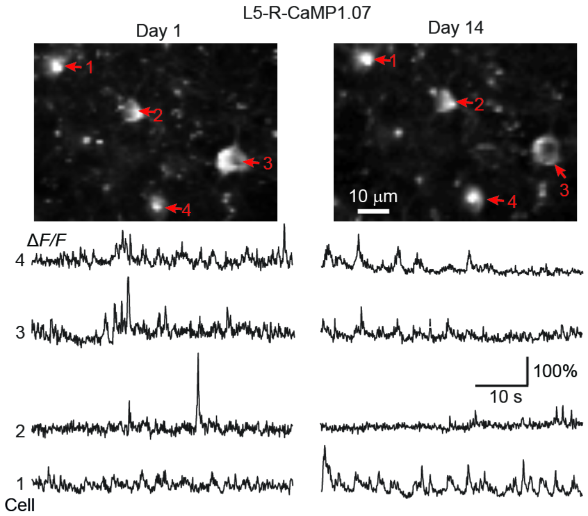

S5 Fig. Repeated functional imaging of R-CaMP1.07 signals from L5 neurons. Top: Two-photon images of example L5 neurons in a L5-R-CaMP1.07 mouse taken two weeks apart. Bottom: Repeatedly measured spontaneous somatic Δ*F/F* calcium signals for the 4 example neurons marked in the images.
